# Editorial: New insight into Huntington's disease: From neuropathology to possible therapeutic targets

**DOI:** 10.3389/fnins.2023.1138712

**Published:** 2023-02-02

**Authors:** Ana María Estrada-Sánchez, George V. Rebec, Laurie Galvan

**Affiliations:** ^1^División de Biología Molecular, Laboratorio de Neurobiología, Instituto Potosino de Investigación Científica y Tecnológica (IPICYT), San Luis Potosí, Mexico; ^2^Program in Neuroscience, Department of Psychological and Brain Sciences, Indiana University, Bloomington, IN, United States; ^3^Sciences Department, Université de Nîmes, Nîmes, France

**Keywords:** mutant huntingtin, acanthocytes, irritable behavior, interneurons, cortex, hypothalamus, calcium imaging

In 1872, George Huntington described an illness that is “confined to certain families… in whose veins the seeds of the disease are known to exist.” That seed was later identified as the expansion of the cytosine-adenine-guanine (CAG) repeats within the huntingtin gene that leads to an expanded glutamine repeat tract in the N-terminal end of the huntingtin protein (Gusella et al., 1983; MacDonald et al., 1993; Huntington, 2003). This illness is known as Huntington's disease and depending on the length of the expanded glutamine tract, it can manifest during childhood or adulthood. In the former, the main phenotypical alterations are body rigidity and epileptic seizures. The presence of psychiatric disturbances, the progressive development of generalized involuntary movements, and dementia characterize the onset of Huntington's disease during adulthood.

Interestingly, nine other neurodegenerative disorders are caused by CAG expansion (Stoyas and La Spada, 2018). Although each disorder has a particular set of phenotypical alterations related to the affected gene, all share common molecular neuropathological mechanisms elicited by the CAG expansion. Among these disorders, Huntington's disease is the most studied, allowing us to understand its neuropathology better and bringing us closer to a possible treatment. Furthermore, these advances have improved our understanding of the common pathological mechanisms between all CAG-related neurodegenerative disorders.

Although the genetic mutation for Huntington's disease was identified 40 years ago, no effective treatment has emerged, partly due to the complexity of the disease. However, multiple efforts are currently being made using new technologies or approaches to clarify these knowledge gaps and allow us to propose new therapeutic strategies. This Research Topic aimed to bring together new advances in understanding Huntington's disease etiology, and here we describe the contribution of each of the research published papers on the Research Topic.

Ubiquitously expressed throughout the body, the huntingtin protein is involved in several key molecular and cellular mechanisms. Therefore, its mutation in Huntington's disease affects genetic, molecular, and cellular functions, mainly in the brain, but the mutation affects other cell types in the body. In this regard, Yu et al. suggest that Huntington's disease patients could also present erythrocytes with irregular surfaces and the presence of several spicules, which is also known as acanthocytosis (Redman et al., 1989). However, these results should be considered cautiously. Peikert et al. raised key methodological issues that make these results premature to conclude that acanthocytosis occurs in Huntington's disease (Peikert et al., 2022). Therefore, more rigorous and detailed follow-up studies are necessary to support this early data collection phase but it could open a new field of research in HD.

In the brain, the huntingtin mutation affects all cell types, including interneurons, which, based on early *postmortem* brain studies, were thought to be relatively spared by the course of the disease (Harper, 1991). Then, the technology advancements, such as transgenic mouse lines and optogenetics, have allowed many laboratories to assess these cellular subpopulations. Consequently, many interneuron dysfunctions have been reported in HD (Cepeda et al., 2013; Mehrabi et al., 2016; Holley et al., 2019). Voelkl et al. thoroughly evaluated cortical GABAergic interneurons in the R6/2 and the zQ175DN knock-in mice models. In the primary motor cortex of the R6/2 model, the authors identified a generalized reduction in cell body size of somatostatin, vasoactive intestinal peptide, and parvalbumin-positive interneurons. In the same model, the primary motor and cingulate cortex showed a selective reduction in somatostatin and vasoactive intestinal peptide markers but not in parvalbumin-positive interneurons, while no changes were observed in the knock-in model of zQ175DN. Indicating that differential cellular changes occur depending on the transgenic models used. However, this finding adds to the growing relevance of cortical alterations in eliciting and shaping the phenotypical alterations of this disorder (Estrada-Sanchez and Rebec, 2013; Estrada-Sanchez et al., 2015, 2019). *Postmortem* brain evaluation of Huntington's disease indicated that patients with more prominent motor signs showed a specific reduction in calbindin-positive interneurons in the primary motor cortex, while patients with mood signs had a loss of calbindin, calretinin, and parvalbumin interneurons in the anterior cingulate cortex (Thu et al., 2010; Kim et al., 2014). Therefore, a region-specific loss of cortical interneurons correlates with the prevalence of motor or mood signs. Thus, it is relevant to study functional changes of cortical interneurons during the progression of the disease, at least in transgenic models, which can be possible with the methodology reviewed by Barry et al.. In this minireview, the authors describe a state-of-the-art method that uses the latest technology to follow cellular circuits (neurons, interneurons, or astrocytes) functioning through endogenously encoded calcium indicators and a miniaturized microscope or miniscope during the progression of the disease. This technology will provide new insights into the progressive functional changes in cortical and striatal neurons, interneurons, and astrocyte microcircuits and its correlation with the development of phenotypical signs in transgenic mouse models of Huntington's disease.

In addition to involuntary motor symptoms, altered body and brain energy metabolism are one of the main pathological features of Huntington's disease. In this sense, pre-symptomatic and symptomatic Huntington's disease patients showed altered peripheral circulating metabolic hormones even before the symptoms appeared, indicating possible alterations in the hypothalamic-pituitary system (Wang et al., 2014). Dickson et al. evaluated hypothalamic transcriptome profiles of overexpression of the wild-type huntingtin gene, mutant huntingtin, or full-length human mutant huntingtin in the BACHD model. Although the transcription profiles are different between the models used, their results indicated alterations in sterol and cholesterol metabolism in the over-expressing wild-type huntingtin gene and mutant huntingtin; in the case of full-length mutant huntingtin, changes in the hypothalamic gene expression differ between young and old BACHD mice, which might be related to the phenotype of this transgenic model. Impaired cholesterol and sterol metabolism emerges as a key alteration in Huntington's disease, as evidenced by the evaluation of postmortem brain samples and transgenic models (for a review, see Kacher et al., 2022). Therefore, these data suggest clinical interventions to modulate cholesterol metabolism, which could positively impact disease progression (Kacher et al., 2022).

Finally, as mentioned above, Huntington's disease patients present psychiatric disturbances, which often occur before the motor signs. Among these alterations, depression, anhedonia, apathy, anxiety, obsessions-compulsions, and irritability are the most commonly reported (Paoli et al., 2017). McLauchlan et al. expand our understanding of irritable and impulsive behavior in patients with Huntington's disease. By combining a set of questionnaires to measure irritability and other tasks that measure provocation, motor inhibition, and decision-making, the authors identified that irritability in a cohort of patients with Huntington's disease is related to an excessive response to provocation. Teasing apart the components that influence irritability is the first step toward increasing the probability of successful therapeutic interventions. In this sense, a recent publication by the same research team, showed that motivational anhedonia (impaired effort for reward) underlies depression in Huntington's disease, which is improved with bupropion treatment and when combined with serotonin reuptake inhibitors elicited a better efficacy to alleviate depression in Huntington's disease (McLauchlan et al., 2022).

Overall, the research articles and the minireview that comprise this Research Topic expand our knowledge on previous topics and open new venues to deepen our understanding of the etiology of Huntington's disease in the hope of identifying new therapeutic targets and future treatments that help patients beyond just alleviating symptoms of the disease.

## Author contributions

AME-S wrote the original draft. GVR and LG reviewed and edited it. All authors approved the final manuscript.

## References

[B1] Cepeda C. Galvan L. Holley S. M. Rao S. P. Andre V. M. Botelho E. P.. (2013). Multiple sources of striatal inhibition are differentially affected in Huntington's disease mouse models. J. Neurosci. 33, 7393–7406. 10.1523/JNEUROSCI.2137-12.2013 23616545 PMC3686572

[B2] Estrada-Sanchez A. M. Blake C. L. Barton S. J. Howe A. G. Rebec G. V. (2019). Lack of mutant huntingtin in cortical efferents improves behavioral inflexibility and corticostriatal dynamics in Huntington's disease mice. J. Neurophysiol. 122, 2621–2629. 10.1152/jn.00777.2018 31693428 PMC6966319

[B3] Estrada-Sanchez A. M. Burroughs C. L. Cavaliere S. Barton S. J. Chen S. Yang X. W.. (2015). Cortical efferents lacking mutant huntingtin improve striatal neuronal activity and behavior in a conditional mouse model of Huntington's disease. J. Neurosci. 35, 4440–4451. 10.1523/JNEUROSCI.2812-14.2015 25762686 PMC4355206

[B4] Estrada-Sanchez A. M. Rebec G. V. (2013). Role of cerebral cortex in the neuropathology of Huntington's disease. Front. Neural Circuits 7, 19. 10.3389/fncir.2013.00019 23423362 PMC3575072

[B5] Gusella J. F. Wexler N. S. Conneally P. M. Naylor S. L. Anderson M. A. Tanzi R. E.. (1983). A polymorphic DNA marker genetically linked to Huntington's disease. Nature 306, 234–238. 10.1038/306234a0 6316146

[B6] Harper P. S. (1991). Huntington's Disease. London: W.B. Saunders Company Ltd.

[B7] Holley S. M. Galvan L. Kamdjou T. Cepeda C. Levine M. S. (2019). Striatal GABAergic interneuron dysfunction in the Q175 mouse model of Huntington's disease. Eur. J. Neurosci. 49, 79–93. 10.1111/ejn.14283 30472747 PMC8320683

[B8] Huntington G. (2003). On chorea. J. Neuropsychiatry Clin. Neurosci. 15, 109–112. 10.1176/jnp.15.1.109 12556582

[B9] Kacher R. Mounier C. Caboche J. Betuing S. (2022). Altered cholesterol homeostasis in Huntington's disease. Front. Aging Neurosci. 14, 797220. 10.3389/fnagi.2022.797220 35517051 PMC9063567

[B10] Kim E. H. Thu D. C. Tippett L. J. Oorschot D. E. Hogg V. M. Roxburgh R.. (2014). Cortical interneuron loss and symptom heterogeneity in Huntington disease. Ann. Neurol. 75, 717–727. 10.1002/ana.24162 24771513

[B11] MacDonald M. E. Ambrose C. M. Duyao M. P. Myers R. H. Lin C. Srinidhi L.. (1993). A novel gene containing a trinucleotide repeat that is expanded and unstable on Huntington's disease chromosomes. The Huntington's Disease Collaborative Research Group. Cell 72, 971–983. 10.1016/0092-8674(93)90585-e 8458085

[B12] McLauchlan D. J. Lancaster T. Craufurd D. Linden D. E. J. Rosser A. E. (2022). Different depression: motivational anhedonia governs antidepressant efficacy in Huntington's disease. Brain Commun. 4, fcac278. 10.1093/braincomms/fcac278 36440100 PMC9683390

[B13] Mehrabi N. F. Waldvogel H. J. Tippett L. J. Hogg V. M. Synek B. J. Faull R. L. (2016). Symptom heterogeneity in Huntington's disease correlates with neuronal degeneration in the cerebral cortex. Neurobiol. Dis. 96, 67–74. 10.1016/j.nbd.2016.08.015 27569581

[B14] Paoli R. A. Botturi A. Ciammola A. Silani V. Prunas C. Lucchiari C.. (2017). Neuropsychiatric burden in Huntington's disease. Brain Sci. 7, 67. 10.3390/brainsci7060067 28621715 PMC5483640

[B15] Peikert K. Storch A. Hermann A. Landwehrmeyer G. B. Walker R. H. Simionato G.. (2022). Commentary: acanthocytes identified in Huntington's disease. Front. Neurosci. 16, 1049676. 10.3389/fnins.2022.1049676 36408380 PMC9673475

[B16] Redman C. M. Huima T. Robbins E. Lee S. Marsh W. L. (1989). Effect of phosphatidylserine on the shape of McLeod red cell acanthocytes. Blood 74, 1826–1835. 2790207

[B17] Stoyas C. A. La Spada A. R. (2018). The CAG-polyglutamine repeat diseases: a clinical, molecular, genetic, and pathophysiologic nosology. Handb. Clin. Neurol. 147, 143–170. 10.1016/B978-0-444-63233-3.00011-7 29325609

[B18] Thu D. C. Oorschot D. E. Tippett L. J. Nana A. L. Hogg V. M. Synek B. J.. (2010). Cell loss in the motor and cingulate cortex correlates with symptomatology in Huntington's disease. Brain 133(Pt 4), 1094–1110. 10.1093/brain/awq047 20375136

[B19] Wang R. Ross C. A. Cai H. Cong W. N. Daimon C. M. Carlson O. D.. (2014). Metabolic and hormonal signatures in pre-manifest and manifest Huntington's disease patients. Front. Physiol. 5, 231. 10.3389/fphys.2014.00231 25002850 PMC4066441

